# Dentistry

**Published:** 1874-04

**Authors:** 


### EDITORIAL, ETC.

*Dentistry.*—From an article published in the *British Journal of Dental Science,* under the title of "The Saturday Review on Dentistry," we take the following:

"To any one who is anxious to prove 'material civilization' a mistake, the inquiry may be suggested, What effect has the invention of knives and forks had upon the teeth of those nations who have condescended to adopt the use of them? For these pernicious utensils plainly render good teeth less a necessary of life than they were before, so that people with bad teeth now survive, transmit their degenerate natural weapons to their descendants, and so on. And, therefore, to Mr. Galton and others, who are anxious to guard the interests of the future by promoting marriage on scientific principles, we may suggest the propriety of including sound teeth in the list of excellences required of those about to marry. The priest or registrar might call upon the parties to a proposed marriage to produce, among other certificates, one showing that they themselves had sound teeth, and likewise their fathers and mothers, their grandfathers and grandmothers, and such other relatives as the *savants* may think a sufficient guarantee against reversion. Or perhaps these matters are to be left to the young people themselves, and a man's asking a girl if she has ever suffered from toothache may one day be a recognized way of hinting that he is coming to the point.

But if such means of securing the peace of posterity fail, others may be devised. Amidst the press of unexpected ideas, projects and events, it is daily becoming more difficult to detect an absurdity at first sight; and so the following ingenious speculation which has been started may yet be realized. What if hereafter every one on arriving at a certain age should be bound to yield up the natural teeth, and receive in exchange an artificial set warranted not to ache, and which might be renewed from time to time in case of their ceasing to fit, being swallowed, stolen by garrotters, or other accidents which already are not unexampled? It would demand some courage perhaps once in a lifetime, but that must be better than once a year as at present? Besides, are there not anæsthetics? And, moreover, stern laws, backed by a public opinion instructed in what truly makes for social welfare, would as in Sparta deter the youth from showing the white feather, and they would sit down smiling; just as some American Indians with whom beards are unfashionable, on reaching the age when rudiments of beards appear, submit, it is said, to have them plucked out hair after hair by squaws, and account it a virtue not to groan or wince, though they are the old squaws who do it. By thus losing all our teeth at once as a matter of course, we should no longer be haunted by the regretful feelings which now disturb us, as wandering about the world, visiting many cities, and perhaps many dentists, we drop them as pledges of mortality, one here and another there, and already long before death reflect that our bones lies scattered on every shore. And this will be a relief to many persons; for how imagination follows those fragments of our being, whoever is familiar with folklore knows well.

We have said, perhaps somewhat hastily, that dentists are our own flesh and blood; but at any rate this acknowledgment does not extend to those dentists who put horrible signs of their profession outside their houses in the public streets. What we refer to is too hideous to describe with decency, but every one must understand us. Such things can only be in place in a scientific museum. The only device to be compared to this one is the pirate's flag with its skull and cross-bones, but the flag is much the less revolting of the two. We should have thought such a mode of advertising would have been considered unprofessional, and we are sure it cannot be attractive."

